# Retraction Note: Molecular insights into the anti-cancer properties of Traditional Tibetan medicine *Yukyung Karne*

**DOI:** 10.1186/s12906-023-03917-w

**Published:** 2023-03-30

**Authors:** Tenzin Choedon, Dawa Dolma, Ganeshan Mathan, Vijay Kumar

**Affiliations:** 1 Virology Group, International Centre for Genetic Engineering and Biotechnology, Aruna Asaf Ali Marg, 110067 New Delhi, India; 2 Department of Biomedical Science, Bharathidasan University, 620024 Tiruchirappalli, India; 3 Tibetan Medical Astro Institute, 176215 Dharamsala, Kangra India

**Retraction Note: BMC Complement Med Ther 14, 380 (2014)**

10.1186/1472-6882-14-380

The Editor has retracted this article. After publication, concerns were raised regarding similar western blot control images in this article and the authors’ later work [1]. The authors explained that the data in the two articles originated from the same experiments. Further checks by the Publisher identified the use of the SKOV6 cell line, which is reported to be contaminated with HeLa cells, making it an unsuitable model for ovarian cancer. This undermines the conclusion that *Yukyung Karne* is specific to ovarian cancer. Due to the cell line issue, the Editor no longer has confidence in the conclusions of this article.

None of the authors agree to this retraction.
